# Dual-energy CT liver fat fraction as prognostic imaging biomarker in critically ill patients

**DOI:** 10.1007/s00330-025-11851-3

**Published:** 2025-08-06

**Authors:** Jennifer Erley, Julia Breckow, Kevin Roedl, Ann-Kathrin Ozga, Alidan Duoerkongjiang, Geraldine de Heer, Niklas Schubert, Fabian Pallasch, Christoph Burdelski, Stefan Kluge, Jin Yamamura, Gerhard Adam, Isabel Molwitz

**Affiliations:** 1 Department of Diagnostic and Interventional Radiology and Nuclear Medicine, University Medical Center Hamburg-Eppendorf, Hamburg, Germany; 2 Department of Intensive Care Medicine, University Medical Center Hamburg-Eppendorf, Hamburg, Germany; 3 Institute of Medical Biometry and Epidemiology, University Medical Center Hamburg-Eppendorf, Hamburg, Germany; 4 Department of Radiology, University Hospital Freiburg, Freiburg, Germany; 5 GE HealthCare, Berlin, Germany

**Keywords:** Computed tomography, Dual-energy, Liver steatosis, Body composition, Critical illness

## Abstract

**Objectives:**

To analyze changes in the liver dual-energy CT fat fraction (liver DECT FF) at the intensive care unit (ICU), and its prognostic value.

**Materials and methods:**

Intubated ICU patients were retrospectively included, who received two clinical DECT (CT1 and CT2) within a minimum interval of 10 days between 11/2019 and 12/2022. The liver DECT FF was determined by combining two regions of interest (ROI) in the right and one ROI in the left liver lobe (minimum area 3.6 cm^2^). The skeletal muscle index, muscle radiodensity attenuation, subcutaneous/visceral adipose tissue area, and waist circumference were assessed. Pre-existing diseases, reasons for ICU admission, ICU scoring systems, and in-hospital mortality were noted. *t*-tests, Wilcoxon tests, linear or Cox regression, Pearson correlation, and intraclass correlation coefficients (ICC) were employed.

**Results:**

Of 76 total patients, 43.4% were female (age 61 ± 12 years) and 97.4% received parenteral nutrition. In-hospital mortality was 60.8%. The liver DECT FF at CT1 was the only parameter associated with in-hospital mortality (hazard ratio: 1.15 [95% confidence interval: 1.07–1.24], *p* < 0.001), and a shorter ICU stay (−3.66 [−6.29 to −1.02] days, *p* < 0.007). DECT FF was not associated with ICU scoring systems. It did not relevantly change within a median of 8 days (3.3 ± 5.5% at CT1; 2.7 ± 4.3% at CT2, *p* = 0.315). Subset inter-observer reproducibility was good (ICC: 0.87 [0.66–0.95]).

**Conclusion:**

The liver DECT FF may serve as an independent prognostic imaging biomarker of mortality in critically ill patients, with a superior value to single-energy body composition parameters.

**Key Points:**

***Question*** *Although the risk of liver steatosis is increased in critically ill patients, it is unknown if liver fat content, when quantified using DECT, is of prognostic value*.

***Findings*** *The liver DECT FF is associated with in-hospital mortality in critically ill patients*.

***Clinical relevance*** *The liver DECT FF may be superior for survival prognosis than single-energy CT body composition parameters of muscle and fat tissue at the ICU*.

**Graphical Abstract:**

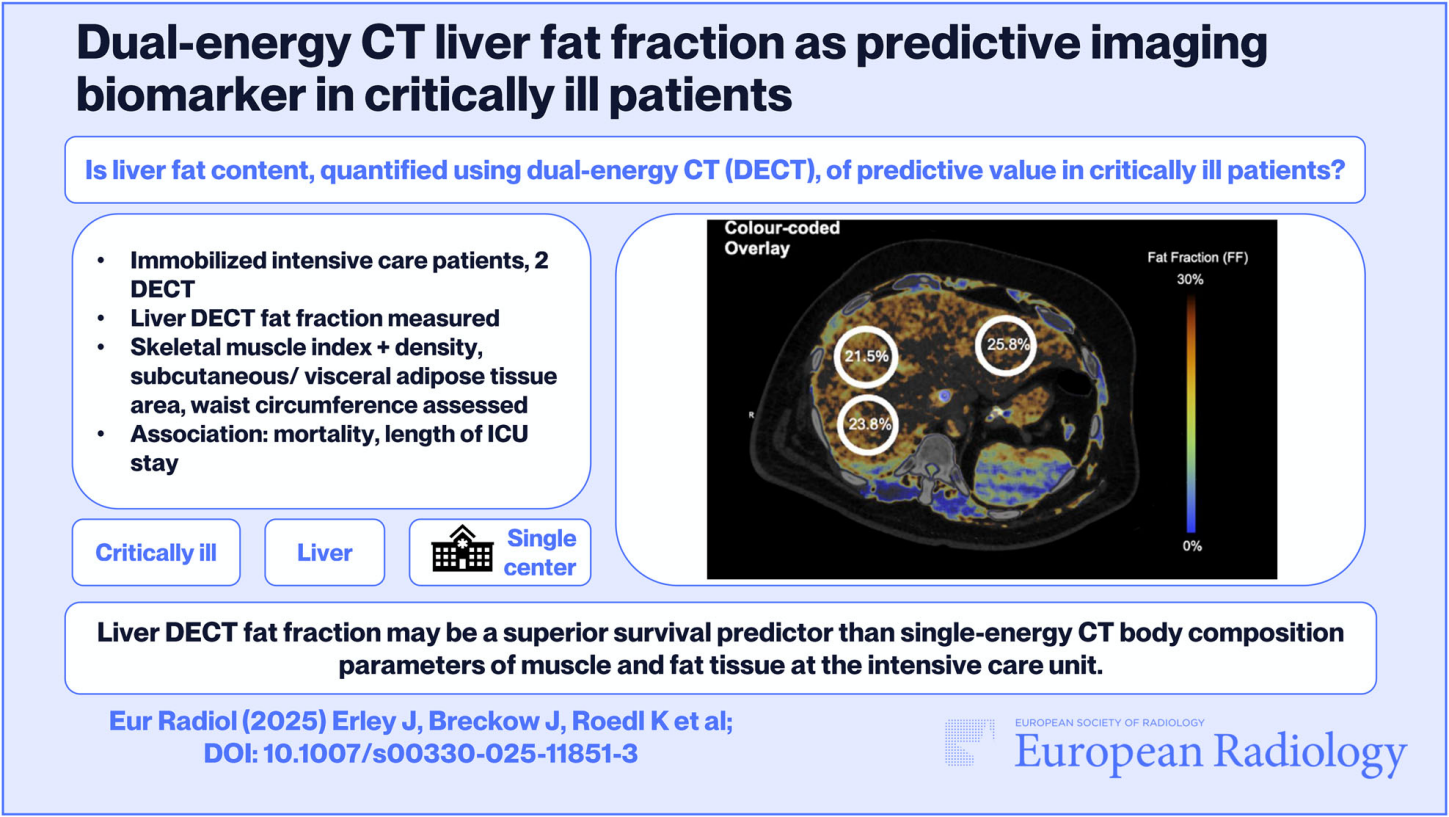

## Introduction

The incidence of liver diseases in the intensive care unit (ICU) has increased significantly, leading to increased morbidity and mortality [1]. Liver steatosis is a fat accumulation in more than 5% of hepatocytes [2]. In critically ill patients, liver steatosis can be primary or acquired, e.g., due to malnutrition [3] or parenteral nutrition [4].

Hepatic function is routinely assessed through laboratory parameters, like bilirubin, in the ICU. However, an increase in these laboratory parameters does not reflect the cause of liver dysfunction, such as liver steatosis. Liver biopsy is the reference standard to assess the etiology of hepatic dysfunction [2]. Due to an increased risk of bleeding and limited diagnostic yield, it is not recommended in critically ill patients. Hence, guidelines recommend imaging to identify subjects with liver steatosis, monitor the onset of fibrosis as a prognosis-determining factor, and predict therapy response [2]. Ultrasound should be performed as a first-line diagnostic test due to its wide availability, low costs, and robust performance. However, ultrasound is operator-dependent with inconsistent accuracy and reliability [2]. Magnetic resonance imaging (MRI), the imaging standard for quantification of liver fat content, is often impossible to perform in ICU patients due to magnetic medical equipment. Computed tomography (CT) also enables the assessment of hepatic steatosis. Additionally, MRI and CT allow the quantification of other nutritional and body composition parameters, such as muscle mass and density, and adipose tissue area. In non-contrast CT scans, established cut-off values for hepatic steatosis (a liver density of 10 Hounsfield Units (HU) less than that of the spleen or an absolute density of less than 40 HU) show excellent specificity compared to histology [5]. However, if a contrast agent is administered, as is necessary for nearly all examinations of critically ill patients, the liver density changes. This reduces the reliability of liver fat quantification [6, 7]. Dual-energy CT (DECT) enables precise material decomposition and quantification by using two X-ray spectra generated with different tube voltages. This enables the quantification of materials with different atomic numbers, such as iodine and fat (the “fat fraction” (FF)), in various organs [8, 9]. Therefore, DECT fat quantification is unbiased by contrast agent.

Despite the increased risk of liver steatosis in critically ill patients, it is unclear if liver fat content changes over time in the ICU. Furthermore, it remains unknown if liver steatosis, quantified using DECT, is of prognostic value. Hence, the goals of this study were to (1) investigate the change in liver DECT FF in a cohort of critically ill immobilized ICU patients, as well as (2) determine the association between liver DECT FF and other CT parameters of body composition/nutritional status with in-hospital mortality, ICU prognostic scores, and length of ICU stay.

## Materials and methods

### Study population

This retrospective observational study was approved by the local ethics committee (Ärztekammer Hamburg, PV7006-4406-BO-ff). A previous analysis of the muscular DECT FF in an overlapping patient cohort was published by Erley et al in European Radiology in 2024 [10]. All analyses were conducted following the Declaration of Helsinki and in compliance with local ethical guidelines. As described previously, inclusion criteria were defined as follows: ICU patients who were (a) intubated (and hence immobilized), and (b) received two contrast-enhanced abdominal DECT scans within a minimum time interval of ten days between November 2019 and December 2022 [10]. Exclusion criteria were (a) age under 18 years, (b) discharge from the ICU between CT scans, and (c) artifacts from metal implants that reduced CT image quality [10]. This manuscript follows the STROBE guidelines [11].

### DECT image acquisition

DECT scans were performed on a dual-source CT scanner. The detailed scanning protocol is depicted in Supplementary Table 1. All subjects received contrast agent, and images were acquired 90 s after injection of 90 mL Iomeprol (Imeron 350 M, Bracco IMAGING).

### Liver DECT FF quantification

**Fig. 1:**
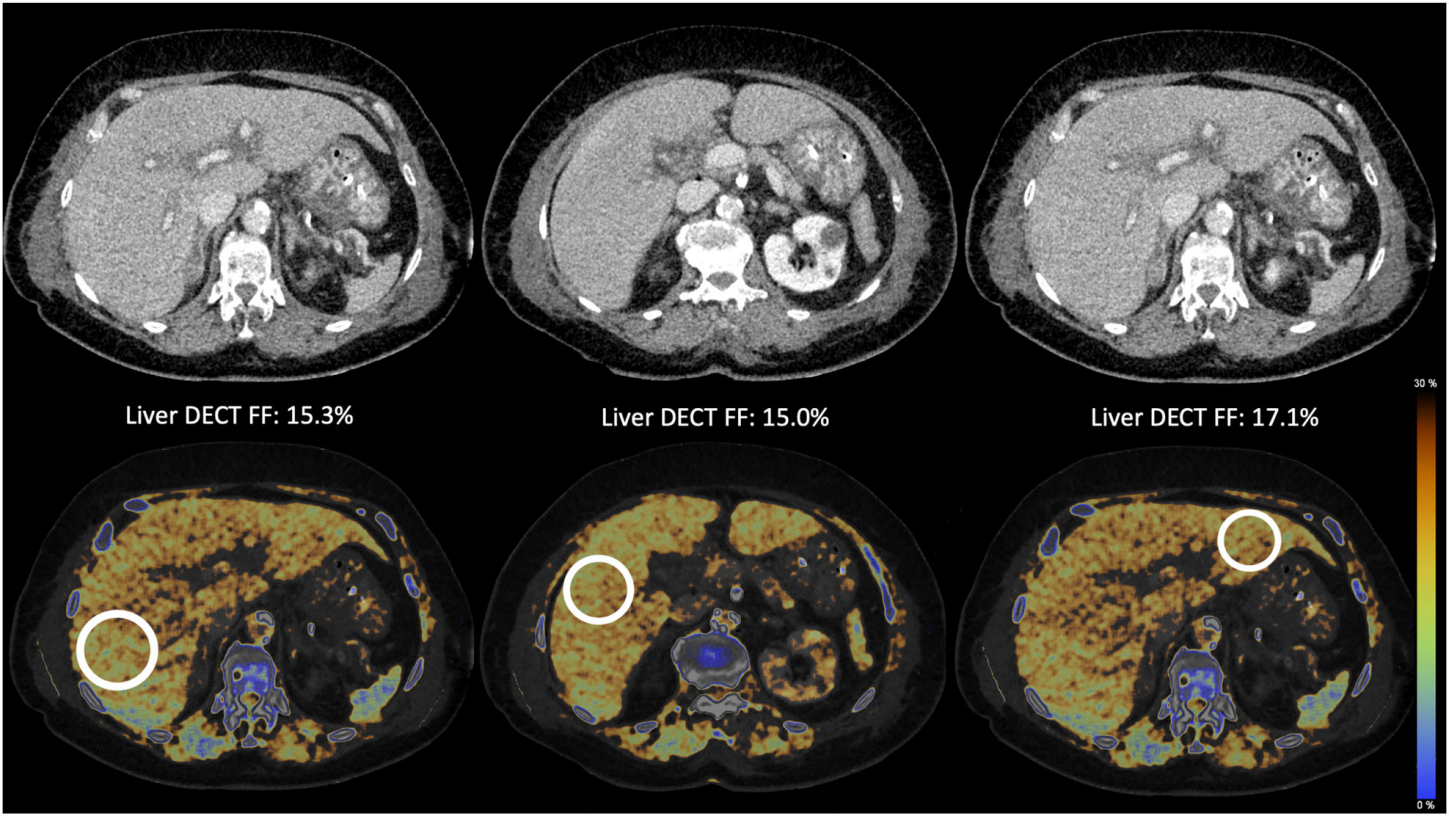
Exemplary liver DECT FF measurements in the right and left liver lobes of a 78-year-old female patient with sepsis. The images illustrate the ROI placed in the color-coded DECT images (two in the right liver lobe and one in the left liver lobe). The ROIs were averaged per patient. This patient showed an overall liver DECT FF of 15.8%, implying liver steatosis

The liver DECT FF was quantified using the commercially available software of the CT scanner’s manufacturer (Syngo.via, Siemens Healthineers). Virtual non-contrast (VNC) images were automatically created using a material decomposition algorithm for soft tissue, iodine, and fat (called “Liver VNC”). The FF was displayed in a color-coded map as an overlay on the DECT images. Two regions of interest (ROI) with a minimum area of 3.6 cm^2^ were drawn into the left liver lobe and one in the right liver lobe, with caution to exclude the liver vasculature or liver lesions, such as cysts. The software automatically calculated the DECT FF within the ROIs. The values for the left and right liver lobes were then averaged to provide the liver DECT FF value for each patient. Figure 1 shows the post-processing steps to analyze the liver DECT FF in an exemplary study patient. Analyses were performed by a radiologist with 4 years of experience. An independent second radiologist with 3 years of experience analyzed a subset of 20 patients to determine inter-observer variability.

### Assessment of conventional CT body composition parameters

An axial CT image at the mid-height of the third lumbar vertebra (L_3_) was exported from the radiological Picture Archiving and Communication system (PACS, GE Centricity), and further processed using the open-source software Image J (National Institutes of Health), according to a step-by-step guide previously published by Gomez-Perez et al [10, 12]. A muscle-specific threshold (−29 to +150 HU) was used to determine the whole skeletal muscle area at the height of L_3_, which was divided by the square body height to calculate the skeletal muscle index (SMI, cm^2^/m^2^) as a measurement of muscle mass. The mean radiation attenuation/density (muscle radiodensity attenuation (MRA), HU), an indirect marker of intramuscular fatty infiltration, was measured for the whole skeletal muscle area. As parameters of the nutritional status, the subcutaneous adipose tissue area (SAT, cm^2^), and visceral adipose tissue area (VAT, cm^2^) were derived using a fat-specific threshold (−150 HU to −30 HU), as was the waist circumference (WC, cm).

### Assessment of clinical parameters

Dates of hospital and ICU admission and dismissal or death were noted. The “Simplified Acute Physiology Score II” (SAPS II) [13], sequential organ failure assessment (SOFA) [14], and the “Charlson Comorbidity Index” (CCI) [15] at ICU admission were calculated. Diseases upon ICU admission were noted and clustered into the following categories: malignancies (solid and hematologic), chronic diseases (e.g., chronic obstructive pulmonary disease, heart failure, and liver disease), chronic-inflammatory diseases (e.g., connective tissue disease), and renal disease (moderate to severe chronic kidney disease), according to the CCI. Mild liver disease was defined as cirrhosis without portal hypertension or chronic hepatitis, moderate liver disease was defined as cirrhosis with portal hypertension, and severe liver disease included patients with variceal bleeding [15]. The reason for ICU admission was determined and categorized (post-surgery vs other medical indications). Laboratory measurements (platelets, lactate, bilirubin, and creatinine) at the time of ICU admission were also noted.

### Statistical analysis

Continuous data are represented using mean ± standard deviation or median [range]. Categorical data are shown via absolute and relative frequencies. Differences in paired CT variables were calculated using a dependent samples *t*-test or the Wilcoxon test, as appropriate, i.e., the Wilcoxon test in case of skewed data. Pearson correlation was assessed. Multivariable linear or Cox regression was employed for prognostic analyses. Model assumptions were checked graphically (histograms (of residuals) for linear models, Schoenfeld residuals, and martingale residuals for the Cox model). The inter-observer variability was determined using intraclass correlation coefficients (two-way mixed, absolute agreement) (ICC). Data on the date of hospital discharge were missing in one patient, and data on in-hospital mortality were missing in two patients. These data were not imputed. No other values were missing. Due to the explorative design of the study, no adjustment for multiple testing was conducted. All *p*-values are thus descriptive. Statistical analyses were conducted using R version 4.2.3 and SPSS (version 28.0.1.1, IBM).

## Results

### Study population

**Fig. 2:**
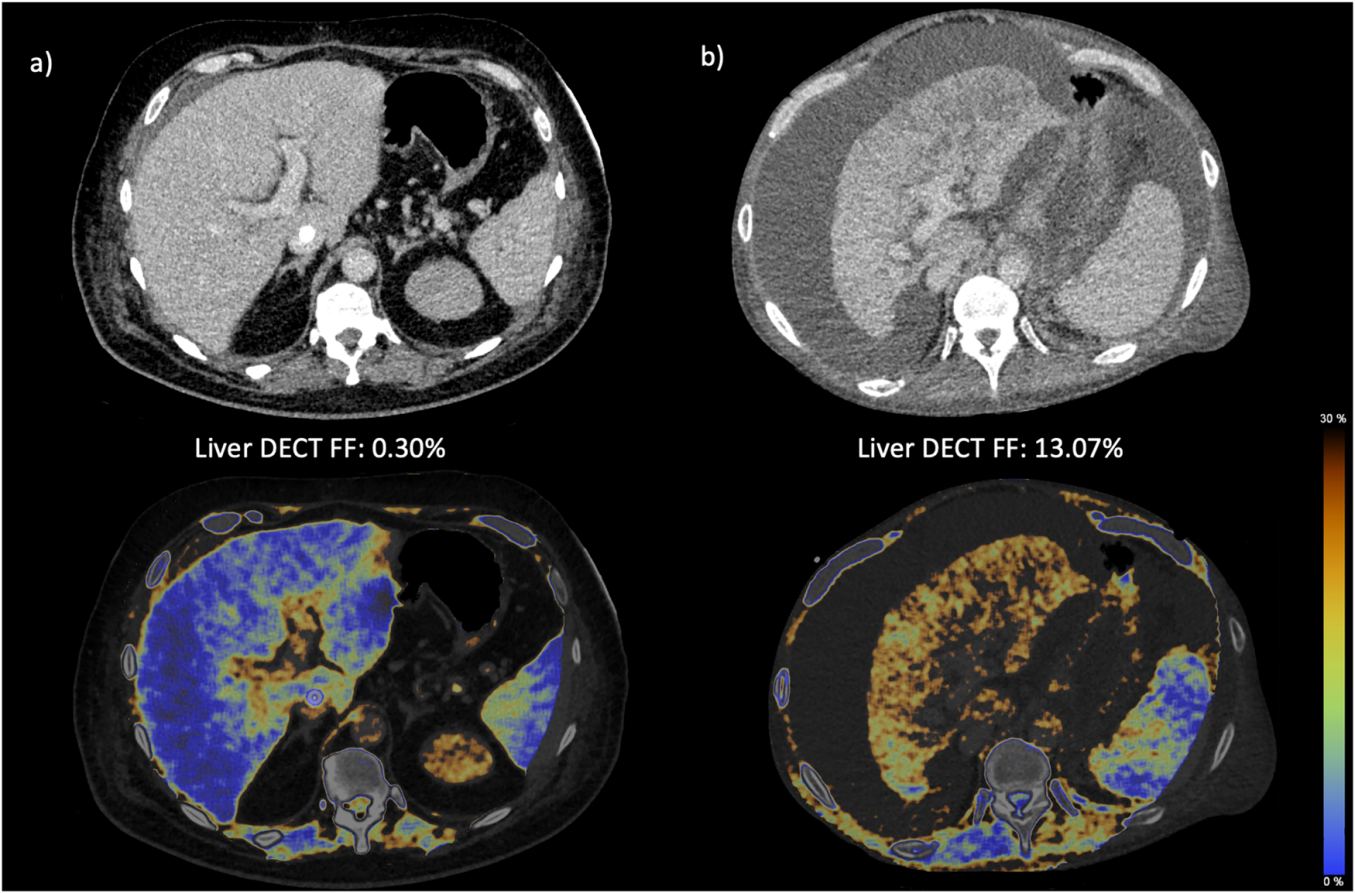
Exemplary contrast-enhanced DECT images and the color-coded overlays to determine the DECT FF in: (**a**) a patient with pneumonia and consecutive organ failure, and (**b**) a patient with decompensated liver cirrhosis due to primary sclerosing cholangitis. The liver DECT FF in patient (**a**) is below the cut-off value for hepatic steatosis. This can also be seen from the blue color-coded overlay and a similar density of liver and spleen. In patient (**b**), the liver appears steatotic with perfusion inhomogeneities, irregular margins, and surrounding ascites. The yellow color-coded overlay demonstrates steatosis, also evident in comparison to the blue overlay of the spleen

The final study collective included 76 patients (33 (43.4%) female, mean age 61 ± 12 years). The median time of ICU stay was 52 [range: 13–321] days. In total, 74 patients (97.4%) received parenteral nutrition, of which the majority (*n* = 73, 96.1%) received both enteral and parenteral nutrition. Two patients (2.6%) only received enteral nutrition, and one patient only received parenteral nutrition. Three patients were clinically malnourished at ICU admission. Liver cirrhosis was known in ten subjects at ICU admission, liver steatosis in one patient, hepatopathy (most likely due to sepsis) in two patients, and two patients had a liver transplant. Of all patients, 37 (48.7%) showed elevated bilirubin levels of 1.2 mg/dL or higher. Liver steatosis (> 5% DECT FF) at CT1 was found in 29 patients (38.2%). Figure 2 depicts two exemplary study patients with varying liver DECT FF. The majority of patients (58%) received CT1 within 10 days after ICU admission. During the hospital stay, 45 patients (60.8%) died. Table 1 illustrates the demographic characteristics and laboratory values of the patient population.

**Table 1:** Demographic characteristics of the patient population

Patient characteristics	
Female, *n* (%)	33 (43.4)
Age (years), mean ± sd	61 ± 12
BMI (kg/m^2^), mean ± sd	25.1 ± 5.2
Time spent in hospital (days), median [range]	65 [14–321]
Time spent in ICU (days), median [range]	52 [13–321]
Time difference between CT1 and CT2, median [range]	21 [10–109]
Time difference between ICU admission and CT1, median [range]	8 [0–175]
Admission post-surgery, *n* (%)	27 (35.5)
Admission for other clinical reasons, *n* (%)	49 (64.5)
SAPS II at admission (points), mean ± sd	44.9 ± 11.5
SOFA at admission (points), mean ± sd	10.1 ± 4.1
CCI at admission (points), mean ± sd	3.5 ± 2.8
In-hospital death, *n* (%)	45 (60.8)
ICU-death, *n* (%)	42 (55.3)

Comorbidities at ICU admission	
Myocardial infarction, *n* (%)	21 (27.6)
Heart failure, *n* (%)	12 (15.8)
Peripheral artery occlusive disease, *n* (%)	9 (11.8)
Cerebrovascular artery occlusive disease, *n* (%)	9 (11.8)
Chronic pulmonary disease, *n* (%)	18 (23.7)
Connective tissue disease, *n* (%)	11 (14.5)
Ulcer, *n* (%)	9 (11.8)
Liver cirrhosis, *n* (%)	10 (13.2)
Liver transplantation, *n* (%)	2 (2.6)
Liver steatosis, *n* (%)	1 (1.3)
Hepatopathy, *n* (%)	2 (2.6)
Moderate or severe liver disease according to CCI, *n* (%)	10 (13.2)
Diabetes mellitus, *n* (%)	6 (7.9)
Renal disease, *n* (%)	18 (23.7)
Malignancies, *n* (%)	26 (34.2)
Chronic diseases, *n* (%)	45 (59.2)
Chronic-inflammatory diseases, *n* (%)	11 (14.5)

ICU treatment regimes	
New renal replacement therapy, *n* (%)	51 (67.1)
Invasive ventilation, *n* (%)	76 (100)
Tracheotomy, *n* (%)	53 (69.7)
Weaning, *n* (%)	25 (32.9)
Vasopressors, *n* (%)	75 (98.7)
Parenteral nutrition, *n* (%)	74 (97.4)
Enteral nutrition, *n* (%)	75 (98.7)
Malnutrition, *n* (%)	3 (3.9)

Laboratory values at ICU admission	
Lactate (mg/dL), median [range]	1.7 [0.3–13.1]
Thrombocytes (10^3^/μL), median [range]	174 [6–806]
Bilirubin (mg/dL), median [range]	1.1 [0.1–22.7]
Creatinine (mg/dL), median [range]	1.5 [0.3–6.0]

*sd* standard deviation, *BMI* body mass index, *ICU* intensive care unit, *SAPS II* simplified acute physiology score II, *SOFA* sequential organ failure assessment, *CCI* Charlson comorbidity index

### Association between liver steatosis and body composition at CT1 and In-hospital mortality

As seen in Supplementary Table 2, the following characteristics were not relevantly associated with liver DECT FF at CT1: sex, age, body mass index (BMI), reason for ICU admission, known chronic/inflammatory/renal/liver diseases, and renal replacement therapy. SMI was influenced by sex and renal replacement therapy, MRA by age, and WC by BMI. SAT also showed an association with BMI, and moderate liver diseases upon admission. VAT was associated with sex, age, and BMI at admission.

The multivariable regression model for mortality prognosis included liver DECT FF/SMI/MRA/WC/SAT/VAT at CT1, age, sex, BMI, and moderate to severe liver disease at admission (Table 2). The liver DECT FF at CT1 was clinically relevantly associated with in-hospital mortality (hazard ratio (HR): 1.15 [95% confidence interval (95% CI): 1.07–1.24], *p* < 0.001), while all other parameters were not. It remained associated with mortality after adjusting the analysis for reasons for ICU admission, renal replacement therapy, ICU prognostic scores, gender, age, and BMI at admission (HR: 1.09 [1.03 to 1.15], *p* = 0.004) (Supplementary Table 3), or when additionally including known liver diseases and malnutrition at admission (HR: 1.06 [1.03 to 1.13], *p* = 0.040 (Supplementary Table 4).

**Table 2:** Results of the Cox regression analysis to determine the association between body composition at CT1, clinical parameters, and in-hospital mortality

Predictors	HR	95% CI	*p*
Liver DECT FF (%)	1.15	1.07–1.24	< 0.001
SMI (cm^2^/m^2^)	0.99	0.94–1.03	0.588
MRA (HU)	0.97	0.94–1.02	0.228
SAT (cm^2^)	1.00	0.99–1.00	0.213
VAT (cm^2^)	0.99	0.99–1.00	0.046
WC (cm)	1.03	0.99–1.07	0.128
Male sex	2.00	0.86–4.65	0.106
Age	1.02	0.99–1.06	0.157
BMI	1.04	0.93–1.16	0.522
Moderate liver disease according to CCI	0.68	0.22–2.07	0.498

The liver DECT-FF was associated with in-hospital mortality. Moreover, the VAT was associated with in-hospital mortality, but according to the HR and 95% CI, of negligible effect

*SMI* skeletal muscle index, *MRA* muscle radiodensity attenuation, *SAT* subcutaneous adipose tissue area, *WC* waist circumference, *BMI* body mass index

### Association between liver DECT FF at CT1 and length of ICU stay

Liver DECT FF at CT1 was also the only body composition parameter clinically relevantly associated with the length of ICU stay. A higher DECT FF was associated with a shorter ICU stay (mean difference with 95% CI: −3.66 [−6.29 to −1.02] days, *p* = 0.007) (Table 3). This association persisted after excluding subjects who died in the ICU (mean difference with 95% CI: −6.04 [−10.70 to −1.38] days, *p* = 0.013). The DECT FF remained the only relevant prognostic factor of the length of ICU stay when additionally including known liver diseases and nutritional status (−3.98 [−6.84 to −1.13] days, *p* = 0.007, Supplementary Table 5). All other clinical parameters showed no relevant influence on the length of ICU stay.

**Table 3:** Results of the linear regression analysis to determine the association between body composition at CT1, clinical parameters, and length of ICU stay

Predictors	Mean difference	95% CI	*p*
Liver DECT FF (%)	−3.66	−6.29 to −1.02	0.007
SMI (cm^2^/m^2^)	0.63	−1.10 to 2.36	0.466
MRA (HU)	−1.12	−2.66 to 0.42	0.152
SAT (cm^2^)	0.08	−0.15 to 0.31	0.491
VAT (cm^2^)	0.02	−0.21 to 0.25	0.864
WC (cm)	−0.31	−1.68 to 1.06	0.649
Male sex	4.32	−29.5 to 38.1	0.799
Age	−0.87	−2.16 to 0.43	0.185
BMI	−1.69	−5.97 to 2.59	0.433
Liver disease	−0.97	−44.73 to 42.79	0.965

Only the liver DECT-FF was associated with the length of ICU stay.

*SMI* skeletal muscle index, *MRA* muscle radiodensity attenuation, *SAT* subcutaneous adipose tissue area, *VAT* visceral adipose tissue area, *WC* waist circumference, *BMI* body mass index, *95% CI* 95% confidence interval

### Association between CT body composition at CT1 and ICU scores

The liver DECT FF did not associate with the SOFA (mean difference with 95% CI: 0.03 [−0.14 to 0.20], *p* = 0.729), CCI (mean difference with 95% CI: 0.02 [−0.10 to 0.13], *p* = 0.791), or SAPS II scores (mean difference with 95% CI: 0.18 [−0.31 to 0.67], *p* = 0.472) at ICU admission (Supplementary Table 6).

### Correlation between liver DECT FF and laboratory values

The correlations of liver DECT FF with lactate (*r* = 0.013, creatinine (*r* = 0.081), and bilirubin (*r* = 0.220) were very weak.

### Change in liver DECT FF and routine body composition parameters from CT1 to CT2

Table 4 shows the change in body composition parameters from CT1 to CT2. The liver DECT FF (3.3 ± 5.5% at CT1 vs 2.7 ± 4.3% at CT2, *p* = 0.315) did not change between CT1 and CT2. Of the other body composition parameters, the SMI as a parameter of muscle mass decreased (35.9 ± 8.8 at CT1 vs 31.5 ± 7.5 at CT2, *p* < 0.001), and the MRA decreased (28.3 ± 10.3 HU at CT1 vs 25.1 ± 10.7 HU at CT2, *p* < 0.001).

**Table 4:** Change in CT body composition parameters from CT1 to CT2

Variable	CT1	CT2	Mean difference^a^ with 95% CI	*p*
Liver DECT FF (%), mean ± sd	3.3 ± 5.5	2.7 ± 4.3	0.65 [−0.63 to 1.93]	0.315
SMI (cm^2^/m^2^), mean ± sd	35.9 ± 8.8	31.5 ± 7.5	4.42 [2.94 to 5.89]	< 0.001
MRA (HU), mean ± sd	28.3 ± 10.3	25.1 ± 10.7	3.19 [1.76 to 4.62]	< 0.001
SAT (cm^2^), median [IQR]	145.9 [57.7–221.3]	141.9 [67.8–201.4]		0.056
VAT (cm^2^), median [IQR]	106.5 [46.4–194.3]	103.4 [42.0–181.4]		0.083
WC (cm), mean ± sd	106.6 ± 14.8	107.1 ± 13.5	−0.54 [−2.68 to 1.59]	0.614

There was a decrease in the SMI as a parameter of muscle mass and a decrease in the muscle radiodensity attenuation (MRA), indicating increasing myosteatosis, between the first (CT1) and the second (CT2) CT scan. The mean time difference between CT1 and CT2 was 21 [range: 10–109] days. For SAT and VAT, a non-parametric equivalent to the *t*-test was used (Mann–Whitney *U*-test), so no mean difference is provided

*sd* standard deviation, *IQR* interquartile range, *DECT FF* dual-energy CT fat fraction, *SAT* subcutaneous adipose tissue area, *VAT* visceral adipose tissue area, *WC* waist circumference, *95% CI* 95% confidence interval

^a^ Only given where *t*-test was applied

### Inter-observer reproducibility

The inter-observer reproducibility for a subset of the liver DECT FF measurements was good to excellent (ICC: 0.87 [0.66–0.95]).

## Discussion

DECT enables non-invasive true quantification of liver fat content, unbiased by contrast agent, as an opportunistic screening tool in clinically indicated CT scans. This mono-centric, retrospective study aimed to analyze the changes in liver DECT FF in the ICU and the association with in-hospital survival, length of ICU stay, and ICU prognostic scores. The results of this study were:

- The liver DECT FF was the only CT parameter associated with in-hospital mortality, but not with ICU prognostic scores.
- No changes were observed in the liver DECT FF between CT1 and CT2.

A higher liver DECT FF at CT1 was associated with in-hospital mortality, while the SMI, MRA, VAT, SAT, and WC were not. These results indicate that, upon ICU admission, hepatic steatosis might be a better prognostic marker of survival than conventional measurements of sarcopenia (such as SMI and MRA) or nutritional status (such as SAT and VAT). This finding could be explained by the association between liver steatosis with chronic inflammation and metabolic syndrome, which, by themselves, contribute to worse outcomes [16–18]. Moreover, subjects with pre-existing liver damage (such as steatosis) are more likely to suffer from primary (liver cirrhosis) or acquired liver dysfunction (due to parenteral nutrition, hypoxic liver injury, or intoxications) [4, 19], which results in high short-term mortality [1, 20, 21]. On the other hand, subcutaneous and visceral adipose tissue have been linked to anti-inflammatory properties in some studies [22]. This led to the controversial “obesity paradox”, describing the association between obesity and favorable prognosis [22–24]. Sarcopenia and myosteatosis are consequences of critical illness [25], while liver dysfunction and the associated comorbidities are predominantly potential causes for ICU admission [26]. Therefore, at the time of ICU admission, liver steatosis might better reflect the inflammatory and metabolic state of critically ill patients than measures of sarcopenia and nutritional status. However, as the liver DECT FF does not seem to change at the ICU, while the muscle mass and density worsen, the other CT parameters or their change over time may become relevant for mortality prediction later on, as indicated by a previous study [27].

At the same time, a higher liver DECT FF was associated with a shorter ICU stay in multivariable analyses, also when excluding subjects who died in the ICU. Its true impact on the length of ICU stay thus warrants further investigation.

The link between liver steatosis and ICU mortality has been described previously. As an example, Janini et al observed that ICU patients with diagnostic criteria for non-alcoholic fatty liver disease showed a higher ICU mortality than patients without liver steatosis [18]. However, the results of this study are of particular interest compared to previous analyses, as this study also includes patients with previously undiagnosed liver steatosis. In fact, only 13.2% of this study cohort admitted to the ICU had known liver cirrhosis, and only 1.3% had known liver steatosis, but we found an increased liver fat content in 38.2% of patients. The true number of patients suffering from liver steatosis and potential liver dysfunction upon ICU admission was, therefore, likely higher. That is also supported by 37 patients (48.7%) showing elevated bilirubin levels of 1.2 mg/dL or above upon ICU admission. Thus, CT could be the key modality to screen for and monitor liver steatosis in critically ill patients, as these patients frequently receive repetitive CT exams for various clinical indications. Because most clinical questions in this patient clientele require the administration of an intravenous contrast agent, the reliability of conventional CT-density-based liver fat quantification is limited [6, 7]. DECT allows for to distinction between materials with different atomic numbers, such as iodine, liver tissue, and fat. As a result, the bias of the iodinated contrast agent on fat measurements can be eliminated. The DECT FF has already been validated for liver fat quantification in numerous studies, including phantoms, laboratory animals, and patients, with a good agreement to MRI, non-contrast CT [28], and histology [28, 29]. Following this first study in critically ill patients, further investigations should be conducted to establish clinical use.

Most study patients received parenteral in addition to enteral nutrition, which is associated with liver steatosis as part of the parenteral nutrition-associated liver disease (PNALD) [30]. Hence, we assumed that the liver DECT FF would increase over time in the ICU, but this was not the case. PNALD was previously predominantly observed in patients with long-term total parenteral nutrition, in pediatric patients, and in cases of overfeeding (particularly when requiring long-term mechanical ventilation) [30–34]. Therefore, the patients investigated here might not have shown a high-risk profile for PNALD, and the time course investigated in this study could be too short for detectable changes in liver fat content to occur. Also, most studies defined PNALD as an increase in liver enzymes [3, 34], and only a few used biopsy and/or imaging. Thus, the true prevalence of liver steatosis as a form of PNALD in ICU patients remains unclear. Further studies are warranted to investigate the dynamics of liver steatosis in different groups of critically ill patients and to assess whether different types of PNALD are associated with specific changes in body composition.

## Limitations

This study is retrospective with a relatively small sample size, a heterogeneous patient cohort, and is based on CT data from one vendor. Thus, the results of this analysis need to be validated in a larger, prospective cohort. Multi-vendor comparisons would be of interest to account for the different types of CT scanners (dual-layer detector, etc.) that generate spectral data. The time differences between ICU admission and CT1 varied. However, as liver DECT FF did not change over time at the ICU, it can be expected that the measurements at CT1 reflect the liver fat content at ICU admission. Moreover, the liver DECT FF was determined at three ROIs within the liver, which may not take focal fatty changes into account, but is coherent with previously published studies in the field [35–37]). Prospectively, more representative liver fat values could be derived by the development of automated liver segmentation tools compatible with spectral data. Finally, liver fat contents can be influenced by certain medications such as steroids, which should be accounted for in prospective studies.

## Conclusion

The liver DECT FF was associated with in-hospital mortality in critically ill patients. It showed a higher prognostic power than conventional CT body composition parameters. As assessed using the DECT FF, liver fat content did not appear to change over time in the ICU. In critically ill patients, the liver DECT FF could be a valuable and independent opportunistic marker of hepatic status and outcome.

## Supplementary information

ELECTRONIC SUPPLEMENTARY MATERIAL

## Abbreviation

95% CI: 95% Confidence interval
BMI: Body mass index
CCI: Charlson comorbidity index
CT: Computed tomography
DECT: Dual-energy CT
FF: Fat fraction
HR: Hazard ratio
ICU: Intensive care unit
MRA: Muscle radiodensity attenuation
MRI: Magnetic resonance imaging
SAPS II: Simplified acute physiology score II
SAT: Subcutaneous adipose tissue area
SMI: Skeletal muscle index
SOFA: Sequential organ failure assessment
VAT: Visceral adipose tissue area
VNC: Virtual non-contrast
WC: Waist circumference

## References

[1] Roedl K, Fuhrmann V (2024) [Liver diseases in the intensive care unit]. Med Klin Intensivmed Notfmed 119:449–457 38937335 10.1007/s00063-024-01157-5

[2] European Association for the Study of the L, European Association for the Study of D, European Association for the Study of O (2016) EASL-EASD-EASO clinical practice guidelines for the management of non-alcoholic fatty liver disease. J Hepatol 64:1388–1402 27062661 10.1016/j.jhep.2015.11.004

[3] Grau T, Bonet A, Rubio M et al (2007) Liver dysfunction associated with artificial nutrition in critically ill patients. Crit Care 11:R10 17254321 10.1186/cc5670 PMC2147066

[4] Lakananurak N, Tienchai K (2019) Incidence and risk factors of parenteral nutrition-associated liver disease in hospitalized adults: a prospective cohort study. Clin Nutr ESPEN 34:81–86 31677717 10.1016/j.clnesp.2019.08.009

[5] Pickhardt PJ, Park SH, Hahn L, Lee SG, Bae KT, Yu ES (2012) Specificity of unenhanced CT for non-invasive diagnosis of hepatic steatosis: implications for the investigation of the natural history of incidental steatosis. Eur Radiol 22:1075–1082 22138733 10.1007/s00330-011-2349-2

[6] Kodama Y, Ng CS, Wu TT et al (2007) Comparison of CT methods for determining the fat content of the liver. AJR Am J Roentgenol 188:1307–1312 17449775 10.2214/AJR.06.0992

[7] Starekova J, Hernando D, Pickhardt PJ, Reeder SB (2021) Quantification of liver fat content with CT and MRI: state of the art. Radiology 301:250–262 34546125 10.1148/radiol.2021204288 PMC8574059

[8] McCollough CH, Leng S, Yu L, Fletcher JG (2015) Dual- and multi-energy CT: principles, technical approaches, and clinical applications. Radiology 276:637–653 26302388 10.1148/radiol.2015142631 PMC4557396

[9] Molwitz I, Campbell GM, Knopp T et al (2024) Fat quantification in dual-layer detector spectral CT: how to handle iron overload, varying tube voltage and radiation dose Indices. PLoS One 19:e0302863 38781228 10.1371/journal.pone.0302863 PMC11115214

[10] Erley J, Roedl K, Ozga AK et al (2024) Dual-energy CT muscle fat fraction as a new imaging biomarker of body composition and survival predictor in critically ill patients. Eur Radiol. 10.1007/s00330-024-10779-4 10.1007/s00330-024-10779-4 PMC11519288 38777903

[11] von Elm E, Altman DG, Egger M et al (2007) Strengthening the reporting of observational studies in epidemiology (STROBE) statement: guidelines for reporting observational studies. BMJ 335:806–808 17947786 10.1136/bmj.39335.541782.AD PMC2034723

[12] Gomez-Perez S, McKeever L, Sheean P (2020) Tutorial: a step-by-step guide (Version 2.0) for measuring abdominal circumference and skeletal muscle from a single cross-sectional computed-tomography image using the National Institutes of Health ImageJ. JPEN J Parenter Enteral Nutr 44:419–424 31617218 10.1002/jpen.1721

[13] Le Gall JR, Lemeshow S, Saulnier F (1993) A new simplified acute physiology score (SAPS II) based on a European/North American multicenter study. JAMA 270:2957–2963 8254858 10.1001/jama.270.24.2957

[14] Vincent JL, Moreno R, Takala J et al (1996) The SOFA (sepsis-related organ failure assessment) score to describe organ dysfunction/failure. On behalf of the Working Group on Sepsis-Related Problems of the European Society of Intensive Care Medicine. Intensive Care Med 22:707–710 8844239 10.1007/BF01709751

[15] Charlson ME, Pompei P, Ales KL, MacKenzie CR (1987) A new method of classifying prognostic comorbidity in longitudinal studies: development and validation. J Chronic Dis 40:373–383 3558716 10.1016/0021-9681(87)90171-8

[16] Gong H, He Q, Zhu L et al (2024) Associations between systemic inflammation indicators and nonalcoholic fatty liver disease: evidence from a prospective study. Front Immunol 15:1389967 38979415 10.3389/fimmu.2024.1389967 PMC11228160

[17] Katsiki N, Kolovou G, Vrablik M (2025) Metabolic dysfunction associated-steatotic liver disease (MASLD) and cardiovascular risk: embrace all facets of the disease. Curr Cardiol Rep 27:19 39804409 10.1007/s11886-024-02181-9

[18] Janini E, Fteiha B, Ramlawi I, Mahamid M (2023) Clinical trajectory and predictors of intensive care unit mortality among nonalcoholic fatty liver disease patients: a retrospective case-control study. J Clin Exp Hepatol 13:218–224 36950493 10.1016/j.jceh.2022.11.010 PMC10025687

[19] Portincasa P, Krawczyk M, Smyk W, Lammert F, Di Ciaula A (2020) COVID-19 and non-alcoholic fatty liver disease: two intersecting pandemics. Eur J Clin Invest 50:e13338 10.1111/eci.13338 PMC7361203 32589264

[20] Arroyo V, Moreau R, Jalan R (2020) Acute-on-chronic liver failure. N Engl J Med 382:2137–2145 32459924 10.1056/NEJMra1914900

[21] Kramer L, Jordan B, Druml W, Bauer P, Metnitz PG, Austrian Epidemiologic Study on Intensive Care ASG (2007) Incidence and prognosis of early hepatic dysfunction in critically ill patients-a prospective multicenter study. Crit Care Med 35:1099–1104 17334250 10.1097/01.CCM.0000259462.97164.A0

[22] Langouche L, Perre SV, Thiessen S et al (2010) Alterations in adipose tissue during critical illness: An adaptive and protective response? Am J Respir Crit Care Med 182:507–516 20442437 10.1164/rccm.200909-1395OC

[23] Gupta R, Maitz T, Behnoush AH et al (2022) Obesity paradox in transcatheter aortic valve implantation? Effect of body-mass index on clinical outcomes in patients undergoing transcatheter aortic valve implantation. Eur J Prev Cardiol 29:e362–e364 36130181 10.1093/eurjpc/zwac215

[24] Mahmoud EIE, Awdallah FF (2023) Observational, prospective, single-center study: should body mass index be added to the scoring criteria of hepatic critically ill patients in the intensive care unit. Am J Med Sci 365:63–72 35718123 10.1016/j.amjms.2022.06.005

[25] Hrdy O, Vrbica K, Kovar M, Korbicka T, Stepanova R, Gal R (2023) Incidence of muscle wasting in the critically ill: a prospective observational cohort study. Sci Rep 13:742 36639540 10.1038/s41598-023-28071-8 PMC9839699

[26] Dong V, Karvellas CJ (2019) Acute-on-chronic liver failure: Objective admission and support criteria in the intensive care unit. JHEP Rep 1:44–52 32039351 10.1016/j.jhepr.2019.02.005 PMC7001553

[27] Erley J, Roedl K, Schubert NF et al (2023) Dual-energy CT muscle fat quantification as a new follow-up parameter and predictor of survival in intensive-care patients European Congress of Radiology 2023. Springer, Vienna

[28] Molwitz I, Leiderer M, Ozden C, Yamamura J (2020) Dual-energy computed tomography for fat quantification in the liver and bone marrow: a literature review. Rofo 192:1137–1153 32911556 10.1055/a-1212-6017

[29] Molwitz I, Campbell GM, Yamamura J et al (2022) Fat quantification in dual-layer detector spectral computed tomography: experimental development and first in-patient validation. Invest Radiol 57:463–469 35148536 10.1097/RLI.0000000000000858 PMC9172900

[30] Guthrie G (2022) Parenteral nutrition associated hepatic steatosis and NAFLD intersect at AMPK. Cell Mol Gastroenterol Hepatol 14:724–725 35810785 10.1016/j.jcmgh.2022.06.005 PMC9421576

[31] Reid C (2006) Frequency of under- and overfeeding in mechanically ventilated ICU patients: causes and possible consequences. J Hum Nutr Diet 19:13–22 16448470 10.1111/j.1365-277X.2006.00661.x

[32] Berlana D (2022) Parenteral nutrition overview. Nutrients 14:4480 10.3390/nu14214480 PMC9659055 36364743

[33] Zalikowska-Gardocka M, Przybylkowski A (2020) Review of parenteral nutrition-associated liver disease. Clin Exp Hepatol 6:65–73 32728621 10.5114/ceh.2019.95528 PMC7380469

[34] Kumpf VJ (2006) Parenteral nutrition-associated liver disease in adult and pediatric patients. Nutr Clin Pract 21:279–290 16772545 10.1177/0115426506021003279

[35] Xu JJ, Boesen MR, Hansen SL et al (2022) Assessment of liver fat: dual-energy CT versus conventional CT with and without contrast. Diagnostics (Basel) 12:708 10.3390/diagnostics12030708 PMC8946969 35328261

[36] Gassenmaier S, Kahm K, Walter SS, Machann J, Nikolaou K, Bongers MN (2021) Quantification of liver and muscular fat using contrast-enhanced dual source dual energy computed tomography compared to an established multi-echo Dixon MRI sequence. Eur J Radiol 142:109845 34271430 10.1016/j.ejrad.2021.109845

[37] Zhu L, Wang F, Wang H et al (2024) Liver fat volume fraction measurements based on multi-material decomposition algorithm in patients with nonalcoholic fatty liver disease: the influences of blood vessel, location, and iodine contrast. BMC Med Imaging 24:37 38326746 10.1186/s12880-024-01215-6 PMC10848342

