# Supplementary material for: Dual-energy CT liver fat fraction as prognostic imaging biomarker in critically ill patients
Source: Eur Radiol. 2025 Aug 6;36(2):1341–50. doi: 10.1007/s00330-025-11851-3 (PMC12953330; doi:10.1007/s00330-025-11851-3)
Supplement: Supplementary file 1 — ELECTRONIC SUPPLEMENTARY MATERIAL [file 330_2025_11851_MOESM1_ESM.pdf]

Dual-Energy CT Liver Fat Fraction as Prognostic Imaging Biomarker in Critically Ill Patients

ELECTRONIC SUPPLEMENTARY MATERIAL

Supplementary Table 1: Details of the DECT protocol.

**Scan Parameter**	
Tube voltage (kV)	100 and 150 with tin filter (CARE Dose4D)
Qual. Ref. mAs	190 and 95
Pitch	0.6
Collimation	0.6
Rotation time (s)	0.5
Contrast agent	90 ml Iomeprol
Delay (s)	90
**Reconstruction Parameter (abdominal soft tissue window)**	
Reconstruction	Iterative Reconstruction (vendor-specific ADMIRE 3)
Reconstructed slice thickness and kernel	1.5 mm with Qr40 and 5 mm with Br40

Abbreviations: DECT = dual-energy CT, mAs = milliampere seconds, kV = kilovolts, cm = centimeter, mm = millimeter, s = seconds

Supplementary Table 2: Results of the regression analysis for the association between demographic and clinical variables with the CT body composition parameters at the first CT scan.

**Variables**	**Mean Difference**	**95% CI**	p
**a) dependent variable: DECT FF**			
Male sex	-1.95	-4.61 to 0.70	0.147
Age	-0.05	-0.16 to 0.07	0.395
BMI	0.13	-0.12 to 0.38	0.305
Post-surgical admission	-1.80	-4.51 to 0.93	0.194
Malignancies	0.14	-2.82 to 3.10	0.926
Chronic diseases	0.26	-2.86 to 3.37	0.871
Inflammatory diseases	2.31	-1.63 to 6.25	0.247
RRT at admission	1.02	-3.21 to 5.25	0.631
Renal diseases at admission	0.56	-2.92 to 4.03	0.750
Moderate liver disease at admission	2.27	-1.76 to 6.30	0.266
**b) dependent variable: SMI**			
Male sex	4.21	0.21 to 8.21	0.039
Age	0.14	-0.03 to 0.31	0.106
BMI	0.26	-0.12 to 0.63	0.171
Post-surgical admission	3.37	-0.74 to 7.47	0.106
Malignancies	-2.72	-7.19 to 1.74	0.228
Chronic diseases	-1.46	-6.14 to 3.23	0.537
Inflammatory diseases	-1.67	-7.61 to 4.26	0.576
RRT at admission	-7.48	-13.85 to -1.11	0.022
Renal diseases at admission	0.43	-4.80 to 5.66	0.870
Moderate liver disease at admission	1.52	-4.56 to 7.59	0.620
**c) dependent variable: MRA**			
Male sex	2.62	-2.12 to 7.36	0.274
Age	-0.25	-0.46 to -0.05	0.015
BMI	-0.39	-0.83 to 0.05	0.083
Post-surgical admission	-1.67	-6.54 to 3.19	0.494
Malignancies	-1.38	-6.68 to 3.91	0.603
Chronic diseases	-4.60	-10.16 to 0.96	0.103
Inflammatory diseases	4.50	-2.54 to 11.53	0.206
RRT at admission	-1.67	-9.22 to 5.88	0.660
Renal diseases at admission	0.11	-6.09 to 6.31	0.972
Moderate liver disease at admission	1.18	-6.02 to 8.38	0.744
**d) dependent variable: WC**			
Male sex	4.03	-1.75 to 9.81	0.169
Age	0.18	-0.06 to 0.43	0.145
BMI	1.62	1.09 to 2.16	<0.001

Post-surgical admission	2.06	-3.87 to 7.99	0.491
Malignancies	1.59	-4.87 to 8.04	0.625
Chronic diseases	5.41	-1.37 to 12.18	0.166
Inflammatory diseases	0.93	-7.65 to 9.51	0.829
RRT at admission	-3.74	-12.95 to 5.47	0.420
Renal diseases at admission	-1.30	-8.86 to 6.26	0.733
Moderate liver disease at admission	6.20	-2.58 to 14.97	0.163
**e) dependent variable: SAT**			
Male sex	-27.39	-61.78 to 7.00	0.117
Age	-1.09	-2.55 to 0.36	0.138
BMI	16.56	13.36 to 19.76	<0.001
Post-surgical admission	-2.29	-37.70 to 33.11	0.898
Malignancies	-5.05	-43.39 to 33.30	0.793
Chronic diseases	-3.30	-43.43 to 36.83	0.870
Inflammatory diseases	-15.69	-66.28 to 34.90	0.538
RRT at admission	-30.70	-85.01 to 23.62	0.263
Renal diseases at admission	-3.90	-48.53 to 40.72	0.862
Moderate liver disease at admission	-52.49	-104.31 to -0.67	0.047
**f) dependent variable: VAT**			
Male sex	66.89	32.33 to 101.44	<0.001
Age	1.62	0.16 to 3.08	0.031
BMI	10.79	7.58 to 14.00	<0.001
Post-surgical admission	1.58	-33.99 to 37.15	0.929
Malignancies	23.31	-15.22 to 61.83	0.231
Chronic diseases	27.84	-12.48 to 68.15	0.173
Inflammatory diseases	6.66	-44.16 to 57.49	0.794
RRT at admission	-7.87	-62.43 to 46.70	0.774
Renal diseases at admission	-21.66	-66.50 to 23.17	0.338
Moderate liver disease at admission	19.17	-23.89 to 71.23	0.465

Abbreviations: DECT FF = dual-energy CT fat fraction, SMI = skeletal muscle index, MRA = mean radiodensity attenuation, SAT = subcutaneous adipose tissue area, VAT = visceral adipose tissue area, WC = waist circumference, BMI = body mass index, RRT = renal replacement therapy, 95% CI: 95% confidence interval.

Supplementary Table 3: Results of the Cox regression analysis to determine the association between DECT FF, clinical variables, and in-hospital mortality.

**Predictors**	**HR**	**95% CI**	p
Liver DECT FF at CT1	1.09	1.03 to 1.15	0.004
Male sex	1.52	0.79 to 2.90	0.208
Age	1.02	0.99 to 1.05	0.299
BMI	0.94	0.87 to 1.03	0.191
SOFA	1.00	0.91 to 1.09	0.924
SPAS II	1.03	0.99 to 1.06	0.142
CCI	1.08	0.91 to 1.28	0.372
Post-surgical admission	0.97	0.44 to 2.13	0.930
Malignancies	0.98	0.40 to 2.43	0.967
Chronic diseases	0.94	0.40 to 2.22	0.890
Inflammatory diseases	3.25	1.29 to 8.19	0.013
RRT	1.20	0.41 to 3.49	0.734

Legend: Only the liver dual-energy CT fat fraction (DECT-FF) at the first CT scan (CT1) and inflammatory diseases were associated with in-hospital mortality.

Abbreviations: HR=hazard ratio, 95% CI = 95% confidence interval, BMI = body mass index, SOFA = Sequential Organ Failure Assessment, SAPS II = Simplified Acute Physiology Score II, CCI = Charlson Comorbidity Index, RRT = renal replacement therapy

Supplementary Table 4: Results of the Cox regression analysis to determine the association between DECT FF, clinical variables, and in-hospital mortality, including liver disease and nutritional status.

**Predictors**	**HR**	**95% CI**	p
Liver DECT FF at CT1	1.06	1.03 to 1.13	0.040
Male sex	1.63	0.85 to 3.15	0.144
Age	1.02	0.99 to 1.05	0.239
BMI	0.97	0.88 to 1.06	0.436
SOFA	1.02	0.93 to 1.13	0.657
SPAS II	1.01	0.97 to 1.05	0.568
CCI	1.17	0.96 to 1.42	0.120
Post-surgical admission	0.56	0.23 to 1.37	0.205
Malignancies	0.81	0.26 to 2.51	0.713
Chronic diseases	1.01	0.41 to 2.47	0.991
Inflammatory diseases	3.23	1.19 to 8.75	0.021
RRT	0.43	0.14 to 1.38	0.157
Liver cirrhosis	0.50	0.14 to 1.79	0.284
Hepatopathy	1.48	0.12 to 18.40	0.759
Malnutrition	2.27	0.41 to 12.59	0.348

Legend: Only the liver dual-energy CT fat fraction (DECT-FF) at the first CT scan (CT1) and inflammatory diseases were associated with in-hospital mortality. Results of the model have to be interpreted with care due to a high number of variables compared to the number of events. Confidence intervals are large for the impact of hepatopathy and malnutrition indicating unspecific estimations, probably due to the low prevalence. The prevalence of liver steatosis and liver transplantation was too low for these variables to be included in the model. As in the model without liver disease / nutritional status, only the liver DECT FF at the time of the first CT scan was associated with in-hospital mortality.

Abbreviations: HR=hazard ratio, 95% CI = 95% confidence interval, BMI = body mass index, SOFA = Sequential Organ Failure Assessment, SAPS II = Simplified Acute Physiology Score II, CCI = Charlson Comorbidity Index, RRT = renal replacement therapy

Supplementary Table 5: Results of the linear regression analysis to determine the association between body composition at CT1, clinical parameters, and length of ICU stay, including different types of liver disease and nutritional status

**Predictors**	**Mean difference**	**95% CI**	p
Liver DECT FF (%)	-3.98	-6.84 to -1.13	0.007
SMI (cm^2/m^2)	0.48	-1.35 to 2.32	0.601
MRA (HU)	-0.84	-2.61 to 0.93	0.344
SAT (cm^2)	0.09	-0.17 to 0.34	0.500
VAT (cm^2)	-0.03	-0.30 to 0.23	0.793
WC (cm)	-0.50	-2.02 to 1.03	0.515
Male sex	5.10	-31.06 to 41.27	0.779
Age	-0.52	-1.95 to 0.92	0.476
BMI	-0.35	-5.35 to 4.66	0.890
Moderate liver disease according to CCI	36.89	-36.08 to 109.86	0.316
Liver cirrhosis	-42.94	-104.05 to 18.16	0.165
Liver transplantation	-50.46	-160.30 to 59.38	0.361
Known liver steatosis	92.01	-48.99 to 233.02	0.196
Hepatopathy	1.46	-92.70 to 95.62	0.975
Malnutrition	-11.39	-88.82 to 66.04	0.769
SOFA	0.82	-3.41 to 5.05	0.699
SAPS II	-0.90	-2.52 to 0.72	0.271
CCI	2.32	-4.08 to 8.72	0.470

Legend: As patient numbers with underlying liver diseases were small, the corresponding results show large confidence intervals and must be interpreted carefully. The liver dual-energy CT fat fraction (DECT FF) was the only relevant prognostic factor of the length of intensive care unit (ICU) stay.

Abbreviations: SMI = skeletal muscle index, MRA = muscle radiodensity attenuation, SAT = subcutaneous adipose tissue area, VAT = visceral adipose tissue area, WC = waist circumference, BMI = body mass index, 95% CI = 95% confidence interval, SAPS II = Simplified Acute Physiology Score II, SOFA = Sequential Organ Failure Assessment, CCI = Charlson Comorbidity Index

Supplementary Table 6: Results of the regression analysis to determine the association between the liver DECT FF and ICU prognostic scores.

Variables	Mean Difference	95% CI	p
**a) Dependent variable: SAPS II**			
Liver DECT FF at CT	0.18	-0.31 to 0.67	0.472
Male sex	-1.57	-6.93 to 3.78	0.560
Age	0.14	-0.09 to 0.37	0.235
BMI	0.36	-0.15 to 0.87	0.164
**b) Dependent variable: SOFA**			
Liver DECT FF at CT	0.03	-0.14 to 0.20	0.729
Male sex	-0.27	-2.16 to 1.61	0.773
Age	-0.09	-0.17 to -0.01	0.036
BMI	0.14	-0.04 to 0.32	0.120
**c) Dependent variable: CCI**			
Liver DECT FF at CT	0.02	-0.10 to 0.13	0.791
Male sex	0.89	-0.39 to 2.17	0.171
Age	0.05	-0.01 to 0.10	0.080
BMI	-0.01	-0.14 to 0.11	0.829

Legend: There was no relevant association of the liver dual-energy CT fat fraction (DECT FF) and the intensive care unit (ICU) prognostic scores.

Abbreviations: SAPS II = Simplified Acute Physiology Score II, SOFA = Sequential Organ Failure Assessment, CCI = Charlson Comorbidity Index, 95% CI: 95% confidence interval.
